# miRNAs in platelet-poor blood plasma and purified RNA are highly stable: a confirmatory study

**DOI:** 10.1186/s13104-018-3378-6

**Published:** 2018-05-04

**Authors:** Dillon C. Muth, Bonita H. Powell, Zezhou Zhao, Kenneth W. Witwer

**Affiliations:** 1 Department of Molecular and Comparative Pathobiology, The Johns Hopkins University School of Medicine, 733 N. Broadway, Miller Research Building Rm 829, Baltimore, MD 21205 USA; 2 Department of Neurology, The Johns Hopkins University School of Medicine, Baltimore, MD USA

**Keywords:** microRNA, Stability, qPCR, Freeze/thaw, Blood processing, Plasma, RNA isolation

## Abstract

**Objective:**

We wished to re-assess the relative stability of microRNAs (miRNAs) as compared with other RNA molecules, which has been confirmed in many contexts. When bound to Argonaute proteins, miRNAs are protected from degradation, even when released into the extracellular space in ribonucleoprotein complexes, and with or without the protection of membranes in extracellular vesicles. Purified miRNAs also appear to present less of a target for degradation than other RNAs. Although miRNAs are by no means immune to degradation, biological samples subjected to prolonged incubation at room temperature, multiple freeze/thaws, or collection in the presence of inhibitors like heparin, can typically be remediated or used directly for miRNA measurements.

**Results:**

Here, we provide additional confirmation of early, well validated findings on miRNA stability and detectability. Our data also suggest that inadequate depletion of platelets from plasma may explain the occasional report that freeze–thaw cycles can adversely affect plasma miRNA levels. Overall, the repeated observation of miRNA stability is again confirmed.

## Introduction

microRNAs (miRNAs) are short (22 nucleotides on average) RNA molecules that contribute to post-transcriptional gene fine-tuning [1, 2] and have aroused attention as potential biomarkers of disease [3–6] in part because of their stability. In cells, mature miRNAs are relatively long-lived [7–10], with estimated half-lives ranging from around 8 h [11] to an astounding 3 weeks in certain quiescent cells [12]. In contrast, messenger RNAs have short half-lives, often of only several minutes long [13]. Outside the cell [14], miRNAs are also highly stable. They resist degradation in plasma kept at room temperature for up to 24 h or frozen and thawed up to eight times [15]. These findings have been confirmed repeatedly [16–21] and are due to the “life-long,” tight association of mature miRNAs with Argonaute (AGO) proteins [9, 22–25]. Without this association, miRNAs are rapidly degraded in the RNase-rich biological compartments with a half-life of seconds [15].

Despite a strong apparent consensus on miRNA stability, there have also been several seemingly contradictory observations. Certain tissue-specific miRNAs in circulation, like miR-1 (muscle) or miR-122 (hepatocytes), have been reported to be more sensitive to freeze/thaw than others [26]. Decreased detection of several plasma miRNAs after freeze/thaw cycles [27] or incubation of plasma at room temperature [28] was recently reported. To help resolve these apparent differences, we re-examined stability of several commonly investigated miRNAs in platelet-rich and platelet-poor plasma after incubation of plasma at 22 °C and after various freeze–thaw cycles. Since platelets—which are exquisitely temperature-sensitive and may be more susceptible to freeze–thaw damage than smaller carriers of miRNA—are not removed from plasma unless several centrifugations or other interventions are performed [29], we hypothesized that the profound influence of platelets may explain apparently contradictory findings in the literature.

## Main text

### Methods

#### Blood processing and plasma treatments

Fresh blood from human donors was obtained under a university-approved protocol (JHU IRB #CR00011400). Blood was collected into 60 mL syringes pre-loaded with 6 mL anticoagulant Acid Citrate Dextrose (ACD) (Sigma Aldrich, St. Louis, MO. Cat #: C3821. Lot #: SLBQ6570 V). Whole blood was centrifuged within 15 min of draw at 1300×*g* for 15 min to pellet blood cells. Supernatant (platelet-rich plasma or PRP) was aliquoted for later use or centrifuged twice at 2500×*g* for 15 min. Supernatant from the final spin was defined as PPP. Three separate aliquots of both PRP and PPP were used for RNA extraction for each of the following conditions: fresh (immediate RNA isolation); one, two, three, four, five, and six freeze–thaw cycles (− 80 to 22 °C), conducted regularly over 4 days and with rapid thaw; and 24-h incubation at 22 °C.

#### RNA extraction

RNA was extracted from 200 µL of plasma using the Exiqon Biofluids kit as described previously [30], including glycogen as carrier. RNA was stored at − 80 °C until use unless otherwise specified.

#### RNA stability experiment

Aliquots of purified plasma RNA were frozen at − 80 °C, then thawed and incubated at room temperature for 7, 3, 1, and 0 days in a “countdown” design as previously described for shorter time periods [31]. For the RNase A control, RNA was completely degraded by addition of equal volume RNase A (ThermoFisher, #EN0531, stock solution 10 mg/mL) prior to reverse transcription.

#### qPCR assays

miRNA stem–loop reverse transcription quantitative PCR [32] was done as previously described [30] but using 384-well plates. Synthetic cel-miR-39-3p (Qiagen, #219610) was included in the reverse transcription master mix as a spike-in. Input into the reverse transcription reaction was normalized by volume (3 µL for plasma RNA). Assays were obtained from Applied Biosystems/Thermo Fisher, Cat. # 4427975: hsa-miR-16-5p (part number 000391), hsa-miR-21-5p (000397), and cel-miR-39-3p (000200). The quantitative step was done by following the manufacturer’s directions for cycling on a QuantStudio 12 K Flex Real-Time PCR System (Thermo Fisher).

#### Data and material availability

All data collected during this study are available upon request. Materials are specified in the relevant methods sections so that researchers wishing to repeat the experiments can use the same reagents. Purified RNA has been used for this study or related studies and is unlikely to be available to other researchers in sufficient quantities.

### Results

#### Stability of miRNA in fresh versus frozen platelet-rich and -poor blood plasma

**Fig. 1:**
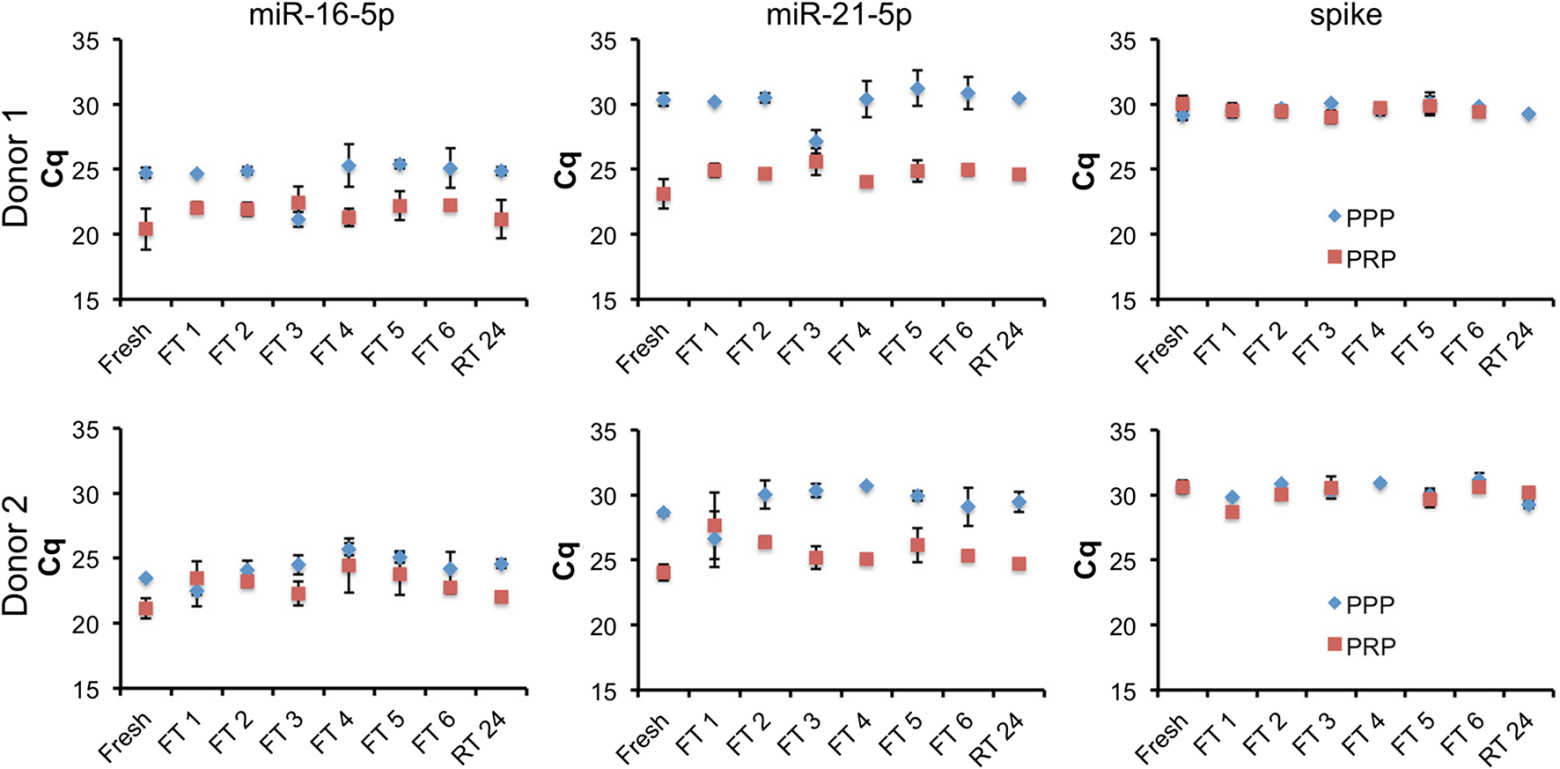
Stability of blood plasma miR-16-5p and miR-21-5p to freeze–thaw and room temperature incubation is associated with removal of platelets. Total RNA was isolated from PRP or PPP immediately after processing (fresh), after one through six freeze–thaw cycles of – 80 to 22 °C (FT 1–6), or after incubation of plasma at 22 °C for 24 h (RT 24). miR-16-5p and miR-21-5p levels as well as a synthetic cel-miR-39-3p spike-in (spike) were assessed by stem-loop/hydrolysis probe qPCR assays, with results presented as Cq. Data are average plus and minus standard deviation for processing replicates. Three processing replicates and three qPCR measurements are included for each condition. No RT and no template reactions were also performed, with all Cq > 37 or undetected (not shown)

Whole blood samples were processed to PRP immediately after draw. PPP was produced from a portion of the PPP. To assess effect of freeze–thaw cycle, PRP and PPP were frozen at − 80 °C and thawed for a total of one through six cycles before RNA isolation. Triplicate aliquots were processed for each condition, and miRs-16-5p and -21-5p were measured by RT-qPCR. As shown in Fig. 1, fresh PRP contained several fold more of both miRNAs than fresh PPP, with the difference attributable to the presence of platelets in the former. Compared with freshly processed plasma (“fresh”), a single freeze–thaw of PRP increased cycle numbers for detection of both miRNAs. Additional cycles of freeze–thaw had no consistent additional effect (Fig. 1, FT 1–6). In contrast, miRNAs in PPP were insensitive to freeze–thaw. Although some technical variability was observed (see especially the outlier points FT3 for donor 1 plasma and FT1 for donor 2, Fig. 1), the average Cq of miRNAs extracted from frozen/thawed PPP was indistinguishable from that of fresh plasma. There was no consistent trend towards higher Cq with higher numbers of freeze–thaw cycles.

#### Stability of miRNA in plasma to incubation at room temperature

In parallel with the freeze–thaw experiments, aliquots of PRP and PPP were incubated at room temperature (approximately 22 °C) for 24 h before RNA isolation. PRP displayed a slight increase in Cq values for both miRNAs after this incubation, although not as pronounced as for the initial freeze–thaw cycle. Similar to the freeze–thaw results, miRNAs in PPP appeared to be insensitive to room temperature incubation (Fig. 1, RT 24).

#### Stability of miRNA in purified RNA

**Fig. 2:**
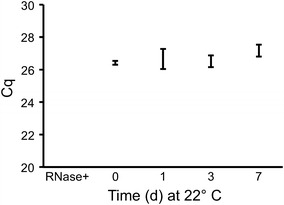
miR-16-5p in purified RNA is stable for up to 1 week at room temperature. Aliquots of total plasma RNA isolated from platelet-poor plasma (different batches from those shown in Fig. 1) were frozen at − 80 °C, then thawed and incubated at room temperature for 0, 1, 3, or 7 days, or thawed and treated with RNase A (Rnase +). qPCR for miR-16-5p was performed. Shown is average ± standard deviation for three experiments

We have previously reported the stability of purified miRNAs in aqueous solution when incubated at room temperature for time periods ranging from 0 to 24 h [31]. We confirmed and extended this observation for miR-16-5p in total plasma RNA purified using the Exiqon Biofluids kit. RNA was incubated at 22 °C for 0, 1, 3, and 7 days prior to qPCR. A slight increase in average Cq was observed after 7 days at room temperature (Fig. 2). However, the range of values at day 7 overlapped with those from other days, and the difference was not significant. Addition of RNase A resulted in complete loss of signal.

### Conclusions

This study re-examined stability of miRNAs in blood plasma, probing abundance of miRNAs in PRP and PPP subjected to eight conditions including freeze–thaw (one to six cycles) and incubation at room temperature for 24 h. Each condition was represented by three processing replicates (for a total of 96 plasma and RNA samples) and three qPCR measurements of RNA from each processing replicate. Like others, we observed no discernible, consistent effect of freeze–thaw or room temperature incubation on miRNA abundance in PPP. In contrast, miRNAs in PRP were affected by one freeze–thaw cycle and, possibly but to a lesser extent, by incubation at room temperature for 24 h. However, freeze–thaw cycles after the initial cycle had no consistent effect on miRNA abundance even for PRP.

These results again reinforce that platelets contain the majority of miRNAs in platelet-rich plasma [29, 33], and that platelets are more susceptible to a single freeze–thaw cycle or incubation at room temperature than smaller miRNA carriers such as exRNPs and EVs that remain after platelet removal. Once damage of platelets occurs in platelet-rich plasma, additional freeze–thaw cycles do not appear to exacerbate miRNA loss; instead, these miRNAs are highly stable [15–21]. While it is possible that different miRNAs would yield different results, this is most likely to occur in PRP, and for miRNAs with different abundance ratios in platelets versus true exRNA fractions. Studies that aim to examine truly extracellular plasma RNA must include careful separation of platelets and their RNA from other RNA sources.

Another potentially important conclusion is that small fold changes in extracellular miRNAs require multiple time points and replicates on which to base any firm conclusions. In this experiment, operators with multiple years of experience with RNA SOPs nevertheless observed anomalous results for several time points or conditions. These results could have led to incorrect conclusions if examined in isolation. For example, the “freeze–thaw 3” condition for PPP (donor 1) and the “freeze–thaw 1” condition for PPP (donor 2) appeared to show an increase in miRNA detection that was not seen for the spiked-in control. The differences we observed were clearly artifactual and introduced at some post-processing or RNA purification step, as a synthetic sequence spiked in just before qPCR was consistently and relatively invariantly detected for all conditions. Without the context of the other conditions/time points, these results might not have been identified as outliers and could have been incorrectly interpreted.

The remarkable stability of miRNAs, both in biological matrices and after RNA purification, has made miRNAs into attractive perceived biomarkers despite their comparative dearth of information content. Protected by a tight association with AGO proteins, mature miRNAs can be detected in biological matrices many years after tissue fixation or freezing, or after weeks or months at above-freezing temperatures, while miRNAs in purified RNA seem to present a smaller “target” for degradation and decay than other RNA species. To be sure, the best practice is always to compare directly only those samples that have been processed and treated in the same way. However, clinical samples and isolated RNA samples alike that have been stored for extended periods, left at room temperatures for hours to days, or subjected to multiple freeze–thaw cycles may still harbor miRNAs with an important tale to tell.

## Limitations

These results were limited to several miRNAs that are commonly studied in circulating biomarker investigations. Other miRNAs, as well as non-miRNA RNA species, may be more or less associated with platelet artifacts.

## Abbreviations

RNA: Ribonucleic acid
miRNA: microRNA
AGO: Argonaute
EV: Extracellular vesicle
qPCR: Quantitative polymerase chain reaction
RNase: Ribonuclease
ACD: Acid citrate dextrose
PRP: Platelet-rich plasma
PPP: Platelet-poor plasma
Cq: Cycle of quantitation
RT: Room temperature
